# Assessing the extent of land-use change around important bat-inhabited caves

**DOI:** 10.1186/s40850-021-00095-5

**Published:** 2021-11-20

**Authors:** Mariëtte Pretorius, Wanda Markotter, Mark Keith

**Affiliations:** 1 Centre for Viral Zoonoses, Department of Medical Virology, Faculty of Health Sciences, University of Pretoria, Pretoria, South Africa; 2 Mammal Research Institute, Department of Zoology and Entomology, Faculty of Natural and Agricultural Sciences, University of Pretoria, Pretoria, South Africa

**Keywords:** Caves, Conservation, Tree-loss, Urbanization, *Miniopterus*, *Rousettus*, South Africa

## Abstract

**Background:**

Modification and destruction of natural habitats are bringing previously unencountered animal populations into contact with humans, with bats considered important zoonotic transmission vectors. Caves and cave-dwelling bats are under-represented in conservation plans. In South Africa, at least two cavernicolous species are of interest as potential zoonotic hosts: the Natal long-fingered bat *Miniopterus natalensis* and the Egyptian fruit bat *Rousettus aegyptiacus*. Little information is available about the anthropogenic pressures these species face around important roost sites. Both bats are numerous and widespread throughout the country; land-use changes and urban expansions are a rising concern for both conservation and increased bat-human contact.

**Results:**

Our study addressed this shortfall by determining the extent of land-cover change around 47 roosts between 2014 and 2018 using existing land cover datasets. We determined the land-cover composition around important roost sites (including maternity, hibernacula and co-roosts), distances to urban settlements and assessed the current protection levels of roost localities. We detected an overall 4% decrease in natural woody vegetation (trees) within 5 km buffer zones of all roost sites, with a 10% decrease detected at co-roost sites alone. Agricultural land cover increased the most near roost sites, followed by plantations and urban land-cover. Overall, roosts were located 4.15 ± 0.91 km from urban settlements in 2018, the distances decreasing as urban areas expand. According to the South African National Biodiversity Institute Ecosystem Threat Status assessment, 72% of roosts fall outside of well-protected ecosystems.

**Conclusions:**

The current lack of regulatory protection of cavernicolous bats and their roosts, increasing anthropogenic expansions and proximity to human settlements raises concerns about increased human-bat contact. Furthermore, uncontrolled roost visitation and vandalism are increasing, contributing to bat health risks and population declines, though the extent of roosts affected is yet to be quantified. In an era where pandemics are predicted to become more frequent and severe due to land-use change, our research is an urgent call for the formal protection of bat-inhabited caves to safeguard both bats and humans.

**Supplementary Information:**

The online version contains supplementary material available at 10.1186/s40850-021-00095-5.

## Background

Globally, caves and other underground openings are vital to the survival of numerous bat (Order Chiroptera) species [9, 21, 52]. Cavernicolous bats are generally colonial and their populations are concentrated in a limited number of large colonies [17, 70]. These bats are typically central-place foragers which concentrate their feeding activities within a relatively small area around the roost site [70]. However, some species may range widely to forage and to reach other roost sites [22] and are therefore sensitive to broader landscape alterations [40]. Threats impacting caves, as well as the foraging area around the roost, make an entire population vulnerable to habitat destruction across a broader scale [69]. Despite this, various caves worldwide are not afforded protection and face threats such as vandalism, pollution, illegal mining and land-cover change [54, 81]. Research has suggested that various bat species, especially those with specific habitat and roosting requirements [39, 74], like cavernicolous bats, are negatively affected by anthropogenically-induced landscape changes [4, 15, 35, 42, 43]. Most bat species are particularly sensitive to the loss of natural woody vegetation [6], land-cover types which are vulnerable to land-use change and fragmentation [43, 47].

South Africa has one of the fastest urbanisation rates worldwide and the demand for resources is increasing [75], leading to negative impacts on the natural landscape and bringing humans and livestock into closer contact with wildlife. The key activities driving habitat loss are land clearing for agriculture, expanding human settlements, intensifying plantation forestry and mining and infrastructure development, jointly resulting in a 21% loss of South Africa’s natural terrestrial ecosystems [80]. Habitat loss is concerning at both a local and a global scale, given that the ongoing modification and destruction of natural habitat and the intensification of anthropogenic land use is bringing previously wild animal populations into closer contact with humans than ever before [37]. Notably, changes in land cover are strongly linked to the increasing emergence of zoonoses worldwide [2], with bats acknowledged as one of the prevalent host species for a variety of viruses [32, 44, 51]. To prevent zoonotic spillover, a variety of ecological interventions (e.g., reducing contact rates between humans and wildlife) can be applied to break the chain of transmission [66]. These ecological approaches necessitate a better understanding of various behavioural, physiological and ecological aspects of the host population for interventions to be successful [82].

South Africa has a rich assemblage of karst caves and other underground openings, however, much of the current focus falls primarily on their archaeological and palaeontological significance [50, 89]. Currently, 22% of 458 ecosystem types in South Africa are classified as threatened [80] and whilst caves may fall within some of these zones, they have not yet been directly assessed as important ecosystems, nor incorporated into active management or conservation plans at a national level that we know of. At least 18% of South Africa’s 60 reported bat species are either fully or partially dependent on caves [46, 61]. Of the cave-dependent species, the Natal-long fingered bat *Miniopterus natalensis* and the Egyptian Rousette bat *Rousettus aegyptiacus* are likely the most numerous and widespread [61]. Both *M. natalensis* and *R. aegyptiacus* distributions appear to be influenced more by the presence of suitable cave roosting sites than broader habitat or climatic associations ([8]; Schoeman et al., 2013). Karst areas (caves/limestone) presented a strong predictive variable in modelled bat species distributions in southern Africa at both a broad [16] and regional [58] scale, highlighting the importance of these landscape features for obligate cave-dwelling species like *M. natalensis* and *R. aegyptiacus* [61]. Currently, out of the known and reported 93 cave localities with bats, there are only 15 caves with verifiable records of resident bats (and 9 confirmed *M. natalensis* roosts) which are currently located within protected areas [67]. The remainder of caves is located outside formally protected areas and vulnerable to land-cover changes and other human impacts and disturbances.

Both *M. natalensis* and *R. aegyptiacus* are species of interest as transmission hosts for potential zoonotic viruses [51], specifically, various potentially zoonotic coronaviruses [30, 31]. The two bat species are characterised by large population sizes and may often be found co-roosting, which increases the chance of cross-species viral sharing and infection [20, 61]. The viral host status and the abundance of *M. natalensis* and *R. aegyptiacus* throughout South Africa raises concerns about an increased chance for contact with humans and livestock. Concurrently, the lack of formal protection for these bats and their obligate roost environment is worrisome for continued cave-dwelling bat survival. This necessitates an understanding of the type and degree of current anthropogenic pressures around known roost sites for these bat species; important both for conservation actions and timely ecological interventions for future zoonotic spillover prevention. Therefore, this study aimed to determine the extent of land-cover change around roosts for *M. natalensis* and *R. aegyptiacus* in South Africa. Specific focus was placed on: 1) determining the extent of land-cover change around roosts with particular focus on the extent of change to intact natural woody vegetation and anthropogenic land-use categories such as agriculture and urbanisation, 2) highlight specific roost site types with the greatest proportions of land cover change and lastly, 3) assess the current ecosystem protection level and threat status of known roost localities.

## Results

**Fig. 1:**
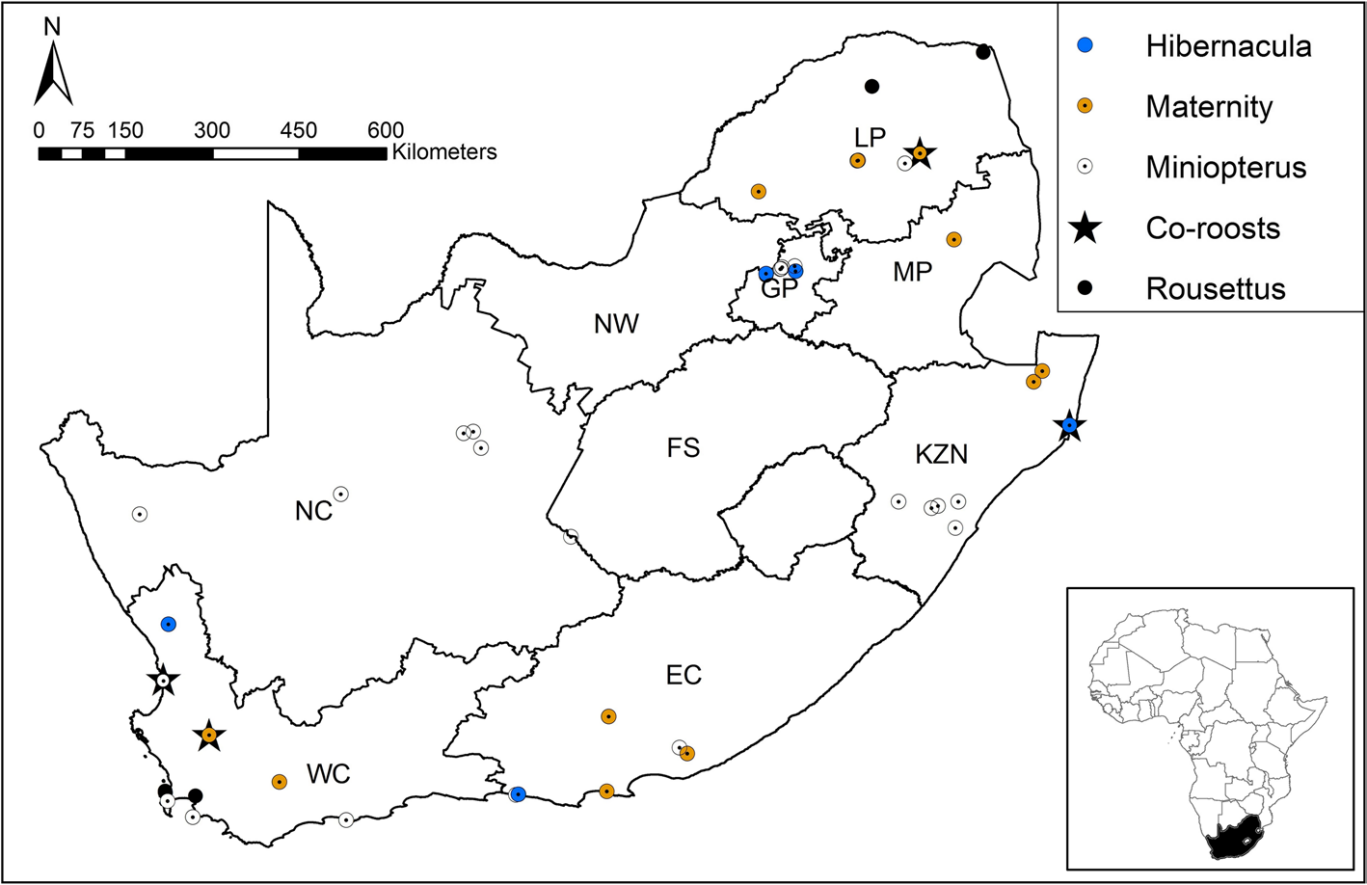
Roosts for Miniopterus natalensis (open circles) and Rousettus aegyptiacus (black circles) throughout South Africa. Important sites (maternity and wintering roosts) for Miniopterus natalensis are indicated in yellow and blue respectively. Locations where both species roost together are indicated by black stars (Co-roosts)

**Fig. 2:**
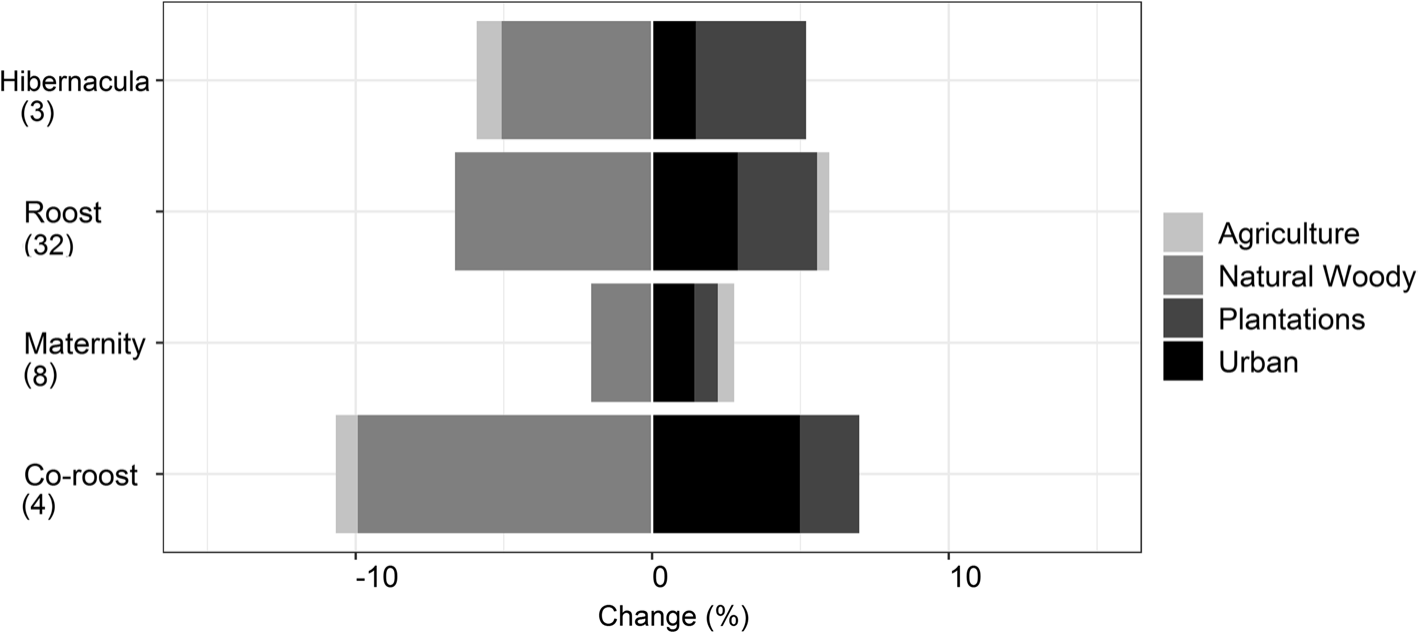
Land-cover change (as a percentage of the total area) between 2014 (in grey) and 2018 (in black) in eight land cover classes within all 5km buffer zones of 47 M. natalensis and R. aegyptiacus roost localities throughout South Africa. Results show the area of each land cover category (in %), values next to bars show the percentage change between 2014 and 2018, negative values are shown in red

**Fig. 3:**
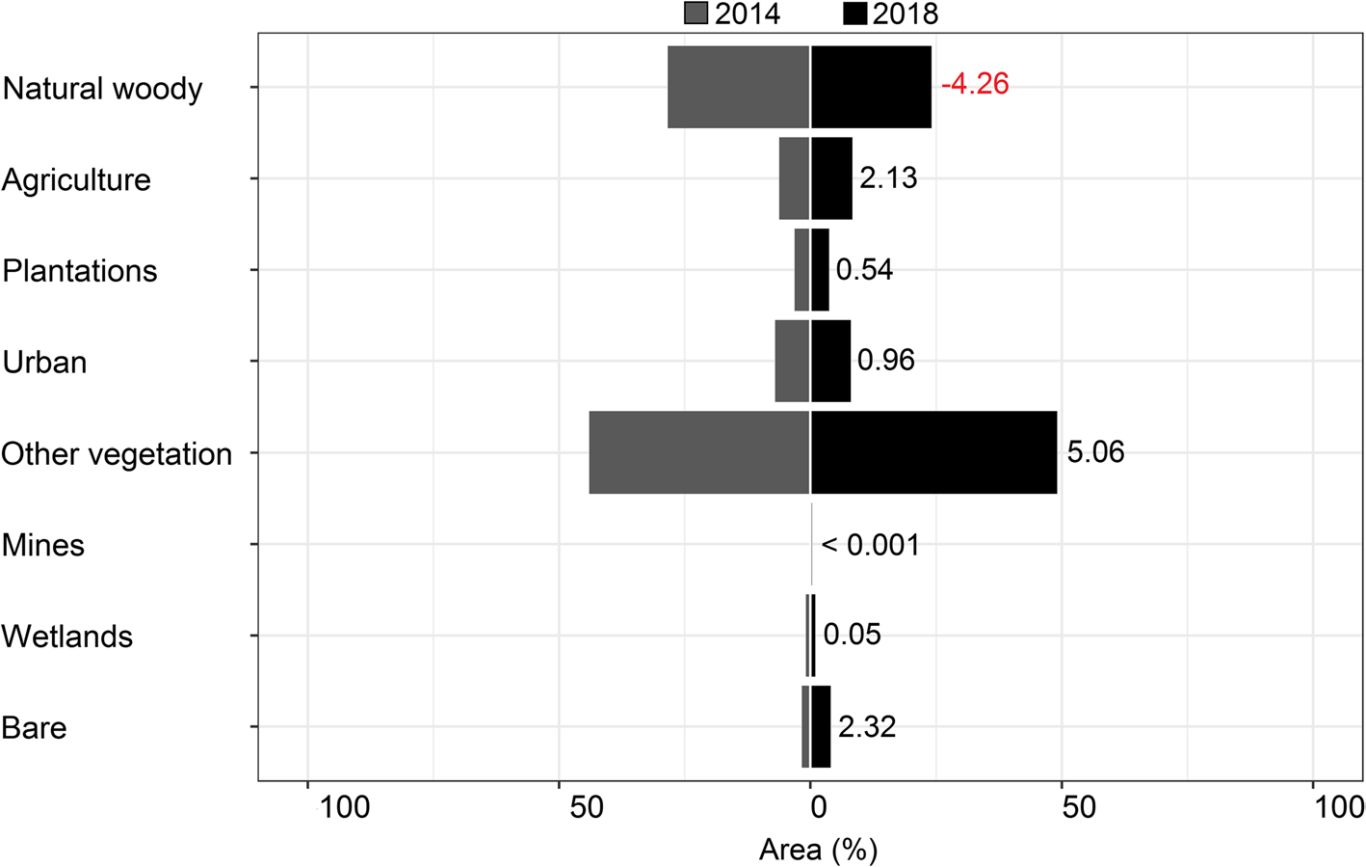
Land-cover change (in percentage) between 2014 and 2018 in three antrhopogenic land cover classes (Agriculture, Plantations and Urban) and Natural woody vegetation (trees) for the four specific roost site types of Miniopterus natalensis (maternity, wintering, roost) and Rousettus aegyptiacus (roost, Co-roost). The number of roosts for each category are shown in parentheses. Loss is shown by negative percentage change

**Fig. 4:**
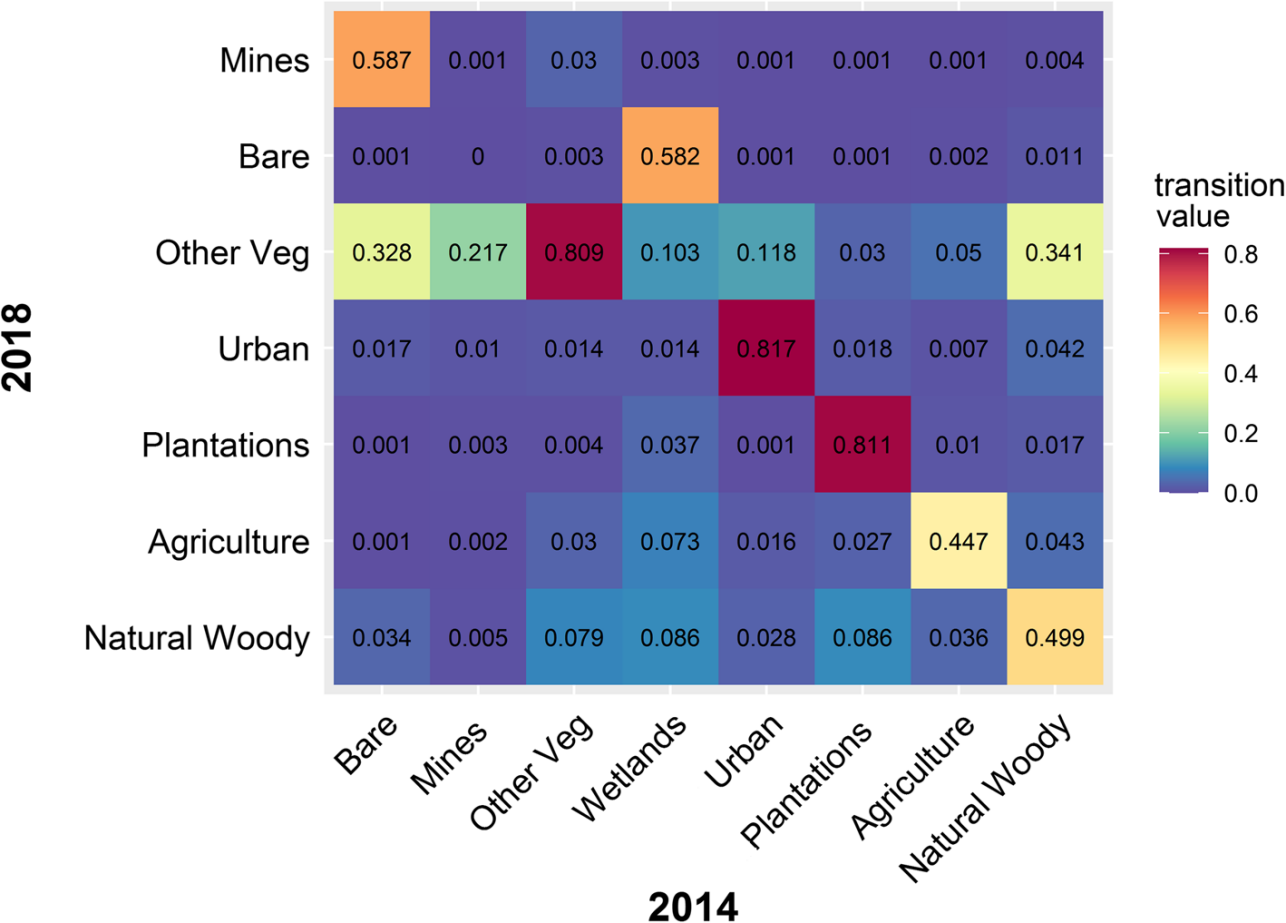
Transition matrix of land cover change for 8 land cover categories around Miniopterus natalensis and Rousettus aegyptiacus roosts throughout South Africa from 2014 to 2018. Higher levels of change is shown on a sliding scale from yellow (>40%) to red (>80%), lower levels of change is shown on a sliding scale from green (> 39%) to blue (> 1%)

**Fig. 5:**
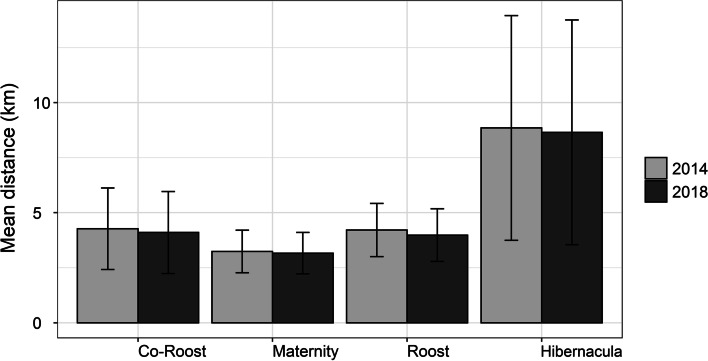
Distance (km) of roost site types for Miniopterus natalensis (Maternity, Wintering, Roost) and Rousettus aegyptiacus (Roost, Co-roost) to the nearest urban settlements in 2014 (light grey) and 2018 (dark grey) throughout South Africa

Within the 5 km buffer zones for all 47 reported roost localities, natural woody vegetation decreased by 4.26% from 2014 to 2018 (Fig. 1). For the anthropogenic land-cover categories, agriculture showed the biggest overall percentage increase, followed by urban land cover and plantations. For the four specific roost types (hibernacula, roost, maternity and co-roosts), natural woody vegetation declined at all four site types but the biggest decline was observed at the co-roost sites (Fig. 2). Urban land-cover increased at all roost site types but also increased the most at the co-roost localities. From 2014 to 2018, the majority of natural woody vegetation loss (~ 34%) resulted from a conversion to the ‘other vegetation’ land cover category, whilst ~ 4% transitioned to urban and agricultural land cover types respectively (Fig. 3). In 2018, the average distance (± SE) for all roosts to the nearest urban settlements were 4.15 ± 0.91 km. This was an average decrease of 0.17 km from 2014 (4.32 ± 0.92 km), although this difference was not statistically significant (W = 1137, *p*-value = 0.91). The distance between urban settlements and roosts decreased at more than half of the roost sites (*n* = 24), with settlements being closer to two roosts by 3 km and 5 km respectively. Hibernacula roosts were located the furthest from urban settlements (8.84 ± 1.27 km), whilst maternity roosts were located the closest (3.43 ± 0.42 km), with distances of all roosts to urban settlements showing a decreasing, although also not statistically significant, trend from 2014 to 2018 (Fig. 4). Seventy-two percent (72%) of all the roost types (*n* = 34) fell within ecosystems that were not protected, poorly protected or moderately protected according to the SANBI 2018 ecosystem protect level assessment, with only 13 roost sites (27%) located within well-protected ecosystems (Fig. 5). The roosts located in well-protected ecosystems included seven roost sites, four maternity sites and two co-roosts.

## Discussion

Land cover around bat-inhabited caves in South Africa has changed and is continuing to change. Within 5 km of known *M. natalensis* and *R. aegyptiacus* cave roosts throughout the country, agriculture, plantations and urban settlements have increased. Simultaneously, natural woody vegetation has declined around all roosts from 2014 to 2018. The majority of change detected for this land-cover type was a conversion to the ‘other vegetation’ category (e.g., to shrubby vegetation or grasses), with conversion to urban and agricultural land-cover being second highest. Trees are a vital component of rural livelihoods throughout South Africa and are harvested for fuelwood and medicine (e.g., medicinal bark) [77, 90]. Current wood harvesting rates in South Africa are unsustainable, with the majority of trees lost within 1.5 km of human settlements [92]. This shifts the vegetation clutter characteristics and dominant vegetation types to shrubby vegetation (< 2 m canopy height) [7, 56], supporting our observations that 34% of natural woody cover around bat roosts shifted to the ‘other vegetation’ category. Trees and tree lines are important to various bat species for foraging and for commuting between sites [25, 45, 55]. The loss of trees also leads to habitat fragmentation, a reduction of bat foraging activity and changes the species composition [24]. Woody vegetation edges are important for clutter-edge foragers, like *M. natalensis* [36] and are likely important linear elements along their migratory routes [67].

Natural woody vegetation loss, and the subsequent loss of food resources (insects [27] and flowers and fruits [12], makes agricultural areas attractive alternatives to insectivorous bats, including *M. natalensis* [86, 91] and frugivorous bats [14]. In particular, *R. aegyptiacus* are attracted to agricultural areas due to the increased availability of fruit crops [13]. The prevalence and extent of crop-feeding by *R. aegyptiacus* bats in South Africa is yet to be assessed [5]. However, the increase in the agricultural land-cover class around cave roost sites from 2014 necessitates future investigation. This is especially important in terms of potential bat-human contact in orchards [23, 53], as well as the damaging long-term impacts on bat health and survival due to pesticide exposure [1, 11, 18, 19].

Urban land cover increased at all four roost site types, with the most change observed at *M. natalensis* and *R. aegyptiacus* co-roost sites. Similarly, expanding urban settlements are getting increasingly closer to more than half of the bat-inhabited caves in South Africa. Urbanisation is one of the greatest threats facing bat populations worldwide [28, 43]. Urban expansion is predicted to cover 1.2 million km^2^ by 2030, with the greatest expansion predicted to occur in biodiverse tropical areas, including Africa [76]. Notably, human populations in major cities in South Africa are projected to increase by as much as 23% by 2030 [62]. Urbanisation generally impacts bat populations negatively through habitat loss, artificial lights (through insect prey loss and commuting disruption) and vehicle collisions on major roads [10, 49, 72]. Both *M. natalensis* and *R. aegyptiacus* appear to be adaptable foragers in urban settings [8, 79], yet little evidence is available as to how these species respond to areas that have recently undergone land-use transformation**.** However, their obligate cave-dwelling natures [61] indicates that their cave roosts sites are critical to their continued survival [16, 58]. Safeguarding co-roosts and maternity roosts may be especially crucial, where reproductive pressures are energetically costly for *M. natalensis* [68], weaken the immune systems of *R. aegyptiacus* during pup weaning periods (Geldenhuys et al., in prep) and where co-roosting may facilitate inter-species infection [20, 51]. At these sites, bat health and survival may be generally compromised and could be exacerbated by human interference.

The majority of cave roosts in this study did not fall within “well-protected” ecosystems [83]. The current lack of regulatory protection for cavernicolous bats and their roosts [59] is leaving South African caves increasingly vulnerable to disturbance and vandalism [34] and these behaviours will increase as more humans live in closer proximity to caves. To enter caves, CPF6 permits from the South African Department of Agriculture And Rural Development are required under the Nature Conservation Ordinance 12 of 1983, Section 9. Vandals are subject to a fine not exceeding ZAR1,500 (approximately $100) or to imprisonment for a period not exceeding 18 months . However, little evidence exists about the enforcement of this rule. Whilst some caves may be considered culturally important under the South African National Heritage Resources Act [No. 25 of 1999], the majority of caves are not reported to be culturally significant and are therefore not included under this act [71]. Increased uncontrolled visitation, graffiti, trampling and cave destruction has led to a visible reduction in the size of *R. aegyptiacus* colonies at a cave roost in the Western Cape Province, with many dead bats observed by researchers throughout the cave [26], even though this cave is located in a protected area. Roosts within protected areas do not automatically guarantee effective conservation protection (as seen with other habitats, see [63]) and cave-specific conservation and protection actions are essential. There is also a need to determine the extent of roost disturbance and destruction for the roost sites mentioned in this study. Though the preservation of natural woody vegetation and forested habitat is also important, the protection of karsts as point resources for cavernicolous bats is essential to the bats’ survival [85] and other cave associated biota.

Anthropogenic activities that cause losses in wildlife habitat quantity and quality increase the opportunities for animal-human interactions and facilitate zoonotic disease transmission [38]. The destruction, disturbance and damage of roosts are some of the main factors affecting bat population decline worldwide [29]. Natural habitat loss, along with human intrusion at roost sites, will likely lead to more frequent human-bat encounters if no steps are taken to formally safeguard cave roost sites in South Africa. This is especially crucial as the worldwide encroachment of growing human populations into wildlife habitats, along with an increase in agriculture and livestock density in areas adjacent to fragmented forests, increases the risk of zoonotic virus transmission from bats to humans [73]. Bats, along with domesticated species, primates and rodents, are a large and diverse order that host a variety of viruses with zoonotic potential [38, 57], with several bat species (including *M. natalensis* and *R. aegyptiacus*) in South Africa earmarked for ongoing monitoring for potentially zoonotic viruses [30, 31, 51]. Our research has highlighted important roost localities for *M. natalensis* and *R. aegyptiacus* that are under pressure from land-cover changes, particularly increasing urbanisation and agricultural activities and the loss of trees. These cave roosts need to be prioritised for future research efforts and conservation actions, particularly given the proximity of some of these sites to human settlements.

## Conclusions

Our study of land cover change around bat-inhabited caves throughout South Africa showed that human impacts are increasing around important bat roost sites; trees have decreased, whilst agriculture and urban settlements have increased. Distances have decreased between settlements and bat roosts. Because many roosts are not located in well-protected ecosystems and no formal cave-conservation plans currently exist, important bat roost sites are at risk of human interference and destruction and the likelihood of bat-human contact throughout South Africa is increasing. These developments are concerning for both human health and continued bat survival and cave-specific conservation is urgently required.

## Methods

**Fig. 6:**
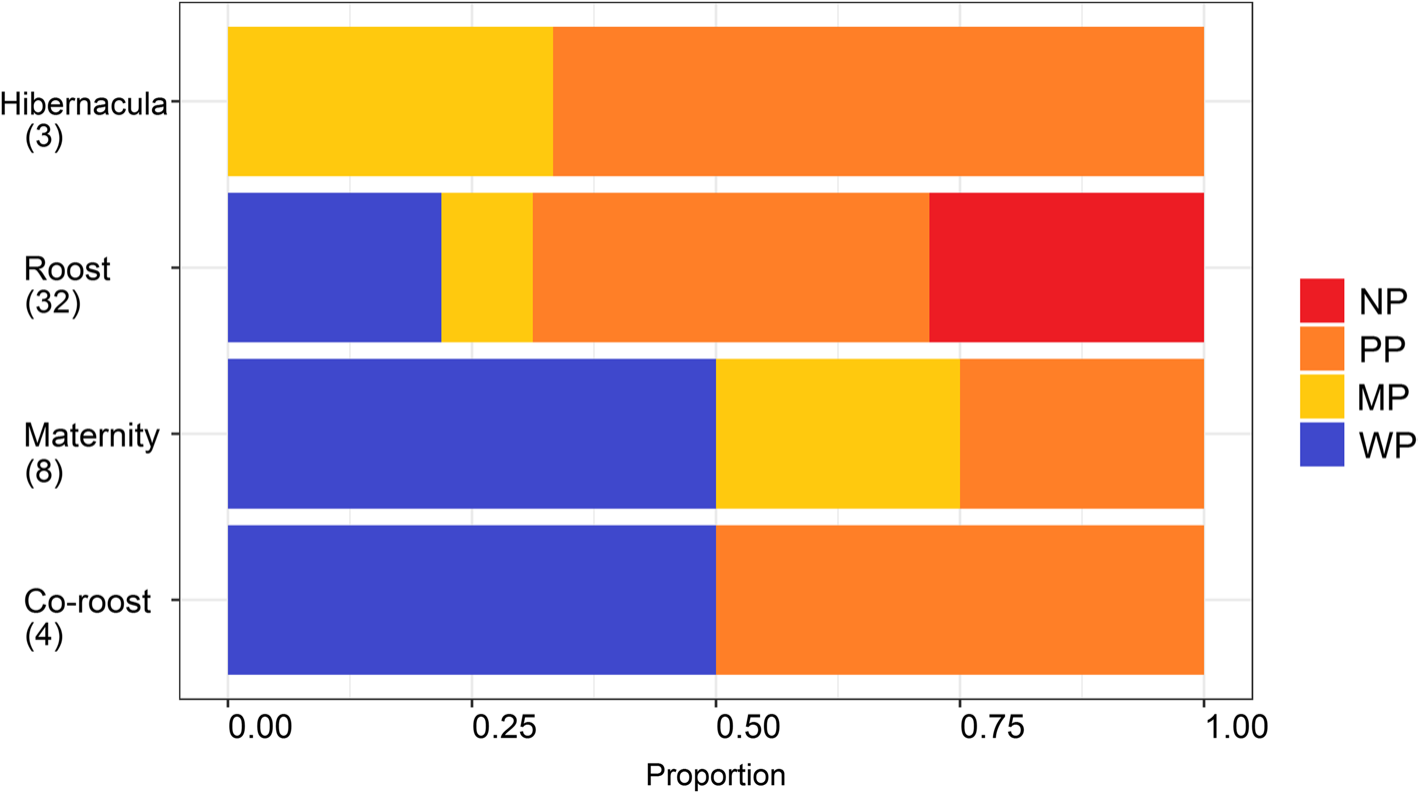
South African National Biodiversity Institute’s five 2018 National Biodiversity Ecosystem Protection Levels (Skowno et al., 2019) for ecosystems where roosts of Miniopterus natalensis and Rousettus aegyptiacus were located. Ecosystems where roosts were located are categorised as not protected (NP, in red), poorly protected (PP, in orange), moderately protected (MP, in yellow) or well protected (WP, in blue). No roosts were detected in hardly protected (HP) ecosystems

We used the cave roost dataset compiled by Pretorius et al. [67], which include 37 *M. natalensis-*specific roosts localities. We expanded this dataset to include 10 *R. aegyptiacus* roost locations and also searched for literature/information mentioning *M. natalensis* and *R. aegyptiacus* in the same roosts (hereafter co-roosts). Roosts were classified as hibernacula roosts (confirmed *M. natalensis* presence April–July, *n* = 3) and maternity (confirmed *M. natalensis* presence October–January, *n* = 10) or simply as ‘roost’ if the importance of the site could not be confirmed [67]. *Rousettus aegyptiacus* roost importance could not be confirmed from literature (except for co-roosts, *n* = 4), therefore locality categories were pooled with the *M. natalensis* roost category (*n* = 32). The dataset used in our analyses comprised 47 confirmed *M. natalensis* and *R. aegyptiacus* roost localities across South Africa (Fig. 6.). See the supplementary material (Table S1) for details about roosts and their related citations. Roost localities were mapped using ArcMap (ArcGIS for Desktop Version 10.5, ESRI Development Team). The home range size of *M. natalensis* and *R. aegyptiacus* are still currently unknown, therefore a 5 km buffer zone was created around each roost and encompasses an average home range for at least two African bat species (see [60, 64]).

South African land-cover datasets for 2014 and 2018 were downloaded from the South African Department of Environmental Affairs (DEA) Environment Geographic Information Systems (EGIS) website (https://egis.environment.gov.za/data_egis/data_download/current). These maps have been created specifically to aid scientific research, environmental planning and protection, economic development, compliance monitoring, enforcement and strategic decision making by providing open access, standardised and comparable reference maps from which landscape changes can be determined and quantified [88]. The 2014 dataset is generated from Landsat 8 imagery acquired from dates spanning 2013–2014 based on 30-m raster cells. The 2018 map represents an updated land cover map currently available for South Africa and is compiled of multi-seasonal 20-m resolution Sentinel 2 satellite imagery [88]. Similar land-cover categories were concatenated to create eight land cover classes for both 2014 and 2018 (see Table 1). These eight land-cover classes for both dates were then exported from each 5 km buffer zone for each cave to Microsoft Excel.

**Table 1:** Eight national land-cover classes and definitions and rationale for inclusion in this study. Only the first four land cover classes were focused on during this study, the remainder of the classes are included for reference

Class	Definition	Rationale
1. Natural woody vegetation	Natural land cover category. 75% or more canopy cover, and canopy heights ranging between 2.5–6 m or more.	Woody vegetation favours bat flight [25, 45, 55], *M. natalensis* classified as a clutter-edge forager [36]
2. Agriculture	Anthropogenic land cover category. Includes commercial and subsistence agriculture and orchards. Active or recently active cultivated lands used for the production of food crops.	Fruit bats are attracted to orchards due to food abundance [14]. Insectivorous species consume pest insect species found in orchards [86, 91]. Often high levels of pesticide use in commercial agriculture sites [41]
3. Plantations	Anthropogenic land cover category. Dense to contiguous cover, planted tree forests, consisting primarily of exotic timber species, with canopy cover exceeding 35%, and canopy heights exceeding 2.5 m. Typically represented by mature commercial plantation tree stands. This class also includes smaller woodlots and windbreaks, where they have been identified by the same spectral-based image modelling procedures used to detect the plantation forests. Includes Open & Sparse Planted Forest and temporary unplanted/clear-felled plantation land cover classes	Single-species plantations may have limited resource value for bats due to lower insect biomass and diversity [3, 65, 94]
4. Urban	Anthropogenic land cover category. Built-up areas containing formally planned and constructed residential structures and associated utilities. The surface is predominantly non-vegetated. This class therefore has the closest spatial representation to all formal residential structures and associated hard-surface footprints. Also included in this category is all other urban structures (recreational fields), informal residential dwellings and villages	Street lights disturb commuting bats [84, 95]. Foraging bat density is lower in urban areas [48]
5. Other vegetation	Natural land cover category. All other vegetation classes with heights below 2.5 m. Includes grassland, low shrubland and sparsely wooded grassland.	
6. Mines	Anthropogenic land cover category. Built-up structures and areas associated with the administration and/or industrial processing and active extraction of mined resources	
7. Wetlands	Natural land cover category. Natural or semi-natural wetlands covered in permanent or seasonal herbaceous vegetation	
8. Bare	Permanent or semi-permanent, natural and anthropogenic non-vegetated surfaces and landfill sites	

Using post-classification methods such as those used by Hardin et al. [33], we calculated the overall area of land-cover/ total area detected within the 5 km buffer zones of all roosts based on the ‘count’ column of both the 2014 and 2018 datasets. To account for the differences in remote sensing technologies (i.e. different number of total sensed pixels) between the two time periods, we compared the differences between percentages of land cover change instead of the area of land-cover change. We also specifically investigated the percentage land cover change around the four different roost types (hibernacula, roost, maternity and co-roost) for the top three anthropogenic land cover types (agriculture, plantations and urban land cover) as well as natural woody vegetation. A land-use transition matrix was created using the overlay functions in ArcGIS system toolbox (see Zhang et al. [96] to represent changes for the land-cover classes around the roosts from 2014 to 2018.

The matrix gives a quantitative description of the current system state and state transition, providing detailed “from-to” change class information [87, 96]. The distances between roosts and the nearest urban settlements between 2014 and 2018 were tested for normality and because the data were not parametrically distributed, we statistically compared the distances using a two-sample Wilcoxon rank-sum test. Lastly, as an additional measure of threat levels to roost sites, we compared roost localities with the [83] National Biodiversity Assessment and Ecosystem Threat Status (NBA-ETS) map (obtained from Biodiversity Geographic Information System website (http://bgis.sanbi.org/SpatialDataset/Detail/2675). Ecosystem types in this dataset are classified according to protection level; not protected (NP), poorly protected (PP), hardly protected (HP), moderately protected (MP) and well protected (WP) [80]. These classifications are based on the proportion of each ecosystem type that remains in a good ecological condition relative to a series of thresholds, using the South African vegetation map, national forest types or high irreplaceability forest patches [80].

Statistical analyses and graphing (using the package *ggplot2* [93]) were performed in R (R Core Team 2017) in RStudio Desktop Software Version 1.1.463.

## Supplementary Information

**Additional file 1: Table S1.** Names and aliases (in parentheses) of *Miniopterus natalensis* and *Rousettus aegyptiacus* roosts acquired from a meta-analysis of websites and scientific literature. The table also shows the roost importance, province where caves occur, the coordinates of the site (Lat, Lon) and the website and associated scientific references. Roost (R) shows *R. aegyptiacus* roost sites, whereas maternity and hibernacula relates to *M. natalensis* only.

## Data Availability

The datasets supporting the conclusions of this article are available in the additional files (Table S1) and from the Figshare repository (10.6084/m9.figshare.14986668).
