# Supplementary material for: Assessing the extent of land-use change around important bat-inhabited caves
Source: BMC Zool. 2021 Nov 20;6:31. doi: 10.1186/s40850-021-00095-5 (PMC8605785; doi:10.1186/s40850-021-00095-5)
Supplement: Supplementary file 1 — Additional file 1: Table S1. Names and aliases (in parentheses) of Miniopterus natalensis and Rousettus aegyptiacus roosts acquired from a meta-analysis of websites and scientific literature. The table also shows the roost importance, province where caves occur, the coordinates of the site (Lat, Lon) and the website and associated scientific references. Roost (R) shows R. aegyptiacus roost sites, whereas maternity and hibernacula relates to M. natalensis only. [file 40850_2021_95_MOESM1_ESM.docx]

**Supplementary Material**

**Table S8.1**: Names and aliases of caves acquired from a meta-analysis of web and scientific literature. Table also shows province where roosts occur, whether each contains *Rousettus aegyptiacus* (Rouset, Y- yes, U- unknown) and *Miniopterus natalensis* (Mini, Y-Yes, U- unknown, N- no), the roost importance (wintering (Mini only), maternity (Mini only) and roost) and websites and scientific literature from which the information was obtained. Roosts marked with * indicate co-roosts for *M. natalensis* and *R. aegyptiacus*

| **Name of Cave/ Alias** | **Province** | **Rouset** | **Mini** | **Importance** | **Website** | **Literature** |
| --- | --- | --- | --- | --- | --- | --- |
| Blombos Cave | Western Cape | N | Y | Roost | 1 | Henshilwood, 2008; Nel & Henshilwood, 2016 |
| Bloukrans Cave | Eastern Cape | N | Y | Maternity | 2 | Herselman & Norton, 1985; Arcus Consultancy Services South Africa (Pty) Limited, 2018 |
| Die Hel A | Western Cape | N | Y | Roost | 3 |  |
| Die Hel B* | Western Cape | Y | Y | Maternity | 3 | Miller-Butterworth et al., 2003 |
| Doornhoek Cave/ Bishopstowe tunnel | KwaZulu-Natal | N | Y | Maternity | 4 | Overal & Wingate, 1976; Staegemann, 2016 |
| Elands Bay Cave 2* | Western Cape | Y | Y | Roost | 5 | Matthews, 1999 |
| Eston Cave | KwaZulu-Natal | N | Y | Roost |  | Laycock, 1973 |
| Fakkel Cave | Gauteng | U | Y | Roost |  | Van der Merwe, 1975 |
| Fawnleas' cave | KwaZulu-Natal | N | Y | Roost |  | Laycock, 1973 |
| Gatkop/ Madimatle/ Sandspruit 1 | Limpopo | N | Y | Maternity | 6 | Van der Merwe, 1973 |
| Gladysvale | Gauteng | N | Y | Wintering |  | Avery, 1995 |
| Goegab/ Goegap Cave | Northern Cape | N | Y | Roost |  | Klein et al., 1991; Avery & Avery, 2011; Salata, 2012 |
| Grahamstown cave/ Howieson's Poort Shelter | Eastern Cape | U | Y | Maternity | 7 | Miller et al., 2013 |
| Hoogland | Gauteng | N | Y | Roost | 8 |  |
| Hopefield Mine | Northern Cape | N | Y | Roost | 9 |  |
| Irene/ Grootboom/ Bakwena | Gauteng | N | Y | Wintering |  | Latham & Herries, 2004; Moir & Durand, 2012 |
| Jozini Dam/ Pongolapoort Dam | KwaZulu-Natal | N | Y | Maternity |  | Miller-Butterworth et al., 2003 |
| Kenhardt Railway Bridge | Northern Cape | N | Y | Roost |  | Avery & Avery, 2011 |
| Koegelbeen Sinkhole | Northern Cape | U | Y | Roost | 10 | Miller-Butterworth et al., 2003 |
| Makapansgat Limeworks | Limpopo | U | Y | Roost | 11 | Schubert et al., 2006; Latham et al., 2007 |
| Mamelodi II | Gauteng | N | Y | Roost |  |  |
| Matlapitsi/ Mahune Cave* | Limpopo | Y | Y | Roost |  | Dietrich et al., 2016; Paweska et al., 2018 |
| Mission Rocks/ Bats Cave* | KwaZulu-Natal | Y | Y | Roost | 12 |  |
| Montagu/ De Hoop Guano Cave | Western Cape | U | Y | Maternity | 13 | McDonald et al., 1990 |
| Peppercorn Cave | Limpopo | N | Y | Maternity | 14 | Van der Merwe, 1975; 1978 |
| **Name of Cave/ Alias** | **Province** | **Rouset** | **Mini** | **Importance** | **Website** | **Literature** |
| Rooiels Cave/ Hangklip Cave | Western Cape | U | Y | Roost | 15 | Plug & Badenhorst, 2001 |
| Schurveberg Cave No I | Gauteng | N | Y | Roost |  | Van der Merwe, 1975;1980 |
| Shongweni Dam | KwaZulu-Natal | N | Y | Roost | 16 | Taylor et al., 1999; Wood, 2012 |
| Soetfontein Cave | Northern Cape | N | Y | Roost |  |  |
| Spioenkop Cave | KwaZulu-Natal | N | Y | Roost |  | Laycock, 1973 |
| Steenkampskraal Mine | Western Cape | U | Y | Wintering | 17 | Plug & Badenhorst, 2001; Goodman et al., 2007 |
| Sudwala Caves/ Sabi Caves/ Kruger Cave | Mpumalanga | U | Y | Maternity |  | Wood, 2012; Mutumi et al., 2016 |
| Table Farm | Eastern Cape | U | Y | Roost | 18 | Bernard, 1985; Wood, 2012 |
| Town Bush Cave | KwaZulu-Natal | N | Y | Roost |  | Laycock, 1973 |
| Tsitsikamma/ Otter Trail Guano Cave/ Storms river mouth Cave | Eastern Cape | N | Y | Roost | 19 |  |
| Uitenhage Mines | Eastern Cape | N | Y | Maternity |  | Bernard & Bojarski 1994; Miller-Butterworth et al., 2003 |
| Vanderkloof Dam | Northern Cape | U | Y | Roost | 20 | Wood, 2012 |
| Wolkberg Cave | Limpopo | N | Y | Roost | 21 |  |
| Wonder Cave Kromdraai | Gauteng | N | Y | Roost | 22 |  |
| Wonderboom/ Apies River Cave | Gauteng | U | Y | Roost |  | Verkerk, 2017 |
| Wonderwerk Cave | Northern Cape | U | Y | Roost | 23 | Beaumont, 2011; Horwitz & Chazan, 2015 |
| Wynberg Cave | Western Cape | Y | U | Roost | 24 | Ferreira et al., 2020 |
| Goro Nature Reserve | Limpopo | Y | U | Roost | 25 | Czenze et al., 2020 |
| Lanner Gorge | Limpopo | Y | U | Roost | 26 | Braack, 1989 |
| Table Mountain Bat Cave | Western Cape | Y | U | Roost | 26 | Barclay et al., 2017 |
| Tsitsikamma Cave | Eastern Cape | Y | U | Roost |  | Herzig-Straschil & Robinson, 1978) |
| Titanic Cave | Western Cape | Y | U | Roost | 26 |  |

**List of websites**

1. https://en.wikipedia.org/wiki/Blombos_Cave
2. http://za.geoview.info/whitchers_cave,940234
3. http://www.darklife.co.za/caves/section-western-cape/groot_winterhoek
4. https://en.wikipedia.org/wiki/Diepkloof_Rock_Shelter
5. http://www.darklife.co.za/caves/section-eastern-cape/tsitsikamma/elands_river_mouth_2_cave
6. https://www.mindat.org/loc-300325.html
7. https://en.wikipedia.org/wiki/Gondolin_Cave
8. https://hoogland.co.za/therapies/nature/bats
9. http://www.cesnet.co.za/assets/16%20Appendix%20G%20Bats%20Scoping%20Study.pdf
10. https://open.uct.ac.za/bitstream/handle/11427/11224/thesis_sci_2012_wood_s.pdf?sequence=1&isAllowed=y; https://xplorio.com/gansbaai/de-kelders-drip-cave-tours/
11. https://en.wikipedia.org/wiki/Makapansgat#Makapansgat_limeworks
12. https://www.zulu.org.za/places-to-go/drakensberg/mhlwazini-cave-P55395
13. https://www.montaguguanocave.co.za/the-cave.php
14. https://en.wikipedia.org/wiki/Makapansgat; http://www.krugerpark.co.za/kruger-park-news-the-greater-mapungubwe-route-25584.html
15. https://www.trekearth.com/gallery/Africa/South_Africa/West/Western_Cape/Rooi-Els/photo1237689.htm
16. https://open.uct.ac.za/bitstream/handle/11427/11224/thesis_sci_2012_wood_s.pdf?sequence=1&isAllowed=y
17. https://open.uct.ac.za/bitstream/handle/11427/11224/thesis_sci_2012_wood_s.pdf?sequence=1&isAllowed=y
18. https://open.uct.ac.za/bitstream/handle/11427/11224/thesis_sci_2012_wood_s.pdf?sequence=1&isAllowed=y
19. http://za.geoview.info/tsitsikamma_guano_cave,1284471p; http://www.darklife.co.za/caves/section-eastern-cape/tsitsikamma/otter_trail_guano_cave
20. https://open.uct.ac.za/bitstream/handle/11427/11224/thesis_sci_2012_wood_s.pdf?sequence=1&isAllowed=y
21. http://www.golimpopo.com/what-to-do/land-activities/wolkberg-cave-system; http://caveandkarst.blogspot.com
22. <https://www.gauteng.net/attractions/wonder_cave>
23. https://en.wikipedia.org/wiki/Wonderwerk_Cave
24. https://lh3.googleusercontent.com/KPk-qdi1Pzj1jrLtN3VvuVwNy0mYPgMcmLpebtL1ByQ1LAkFLdd3zvM98jn03WbcwmIlBbA6Sv3QM4I=w1280-h1024-no
25. <https://en.wikipedia.org/wiki/Gondolin_Cave>
26. https://open.uct.ac.za/bitstream/handle/11427/11224/thesis_sci_2012_wood_s.pdf?sequence=1&isAllowed=y; https://xplorio.com/gansbaai/de-kelders-drip-cave-tours/

**Scientific literature**

Arcus Consultancy Services South Africa (Pty) Limited. 2018. *Pre-Construction Bat Monitoring: Highlands Wind Energy Facilities, Eastern Cape Province Final Environmental* Impact Assessment Report. Available from: <https://arcusconsulting.co.za/wp-content/uploads/sites/2/2019/01/5-Bat-Impact-Assessment.pdf>. Accessed 07/07/2019

Avery, D. M. 1995. A preliminary assessment of the micromammalian remains from Gladysvale Cave, South Africa. *Palaeontologica Africana*, 32:1-10

Avery, D. M. & Avery, G. 2011. Micromammals in the Northern Cape Province of South Africa, past and present. *African Natural History*, 7: 09–39.

Barclay, R. M., Jacobs, D. S., Harding, C. T., McKechnie, A. E., McCulloch, S. D., Markotter, W., ... & Brigham, R. M. (2017). Thermoregulation by captive and free-ranging Egyptian rousette bats (Rousettus aegyptiacus) in South Africa. *Journal of Mammalogy*, *98*(2), 572-578

Beaumont, P. B. 2011. The edge: more on fire-making by about 1.7 million years ago at Wonderwerk Cave in South Africa. *Current Anthropology*, 52: 585–595.

Bernard, R. 1985. Reproduction in the Cape horseshoe bat (*Rhinolophus capensis*) from South Africa. *African Zoology*, 20: 129–135.

Bernard, R. T. F., & Bojarski, C. 1994. Effects of prolactin and hCG treatment on luteal activity and the conceptus during delayed implantation in Schreibers' long-fingered bat (*Miniopterus schreibersii*). *Reproduction*, 100(2), 359-365.

Braack, L. E. O. (1989). Arthropod inhabitants of a tropical cave ‘island’environment provisioned by bats. *Biological Conservation*, *48*(2), 77-84.

Czenze, Z. J., Naidoo, S., Kotze, A., & McKechnie, A. E. (2020). Bat thermoregulation in the heat: Limits to evaporative cooling capacity in three southern African bats. *Journal of thermal biology*, *89*, 102542.

Dietrich, M., Tjale, M. A., Weyer, J., Kearney, T., Seamark, E. C., Nel, L. H., Monadjem, A. & Markotter, W. 2016. Diversity of *Bartonella* and *Rickettsia* spp. in bats and their blood-feeding ectoparasites from South Africa and Swaziland. *PLoS One*, 11: e0152077.

Ferreira, R. L., Giribet, G., Du Preez, G., Ventouras, O., Janion, C., & Silva, M. S. (2020). The Wynberg Cave System, the most important site for cave fauna in South Africa at risk. *Subterranean Biology*, *36*, 73.

Goldberg, P., Miller, C. E., Schiegl, S., Ligouis, B., Berna, F., Conard, N. J. & Wadley, L. 2009. Bedding, hearths, and site maintenance in the Middle Stone Age of Sibudu Cave, KwaZulu-Natal, South Africa. *Archaeological and Anthropological Sciences*, 1: 95–122.

Goodman, S., Ryan, K., Maminirina, C., Fahr, J., Christidis, L. & Appleton, B. 2007. Specific status of populations on Madagascar referred to *Miniopterus fraterculus* (Chiroptera: Vespertilionidae), with description of a new species. *Journal of Mammalogy*, 88: 1216–1229.

Henshilwood, C. S. 2008. *Holocene prehistory of the southern Cape, South Africa: excavations at Blombos Cave and the Blombosfontein Nature Reserve.* Archaeopress Oxford.

Herselman, J. & Norton, P. 1985. The distribution and status of bats (Mammalia: Chiroptera) in the Cape Province. *Annals of the Cape Provincial Museums (Natural History)*, 16: 73–126.

Herzig-Straschil, B., & Robinson, G. A. (1978). On the ecology of the fruit bat, Rousettus aegyptiacus leachi (A. Smith, 1829) in the Tsitsikama Coastal National Park. *Koedoe*, *21*(1), 101-110.

Horwitz, L. K. & Chazan, M. 2015. Past and present at Wonderwerk Cave (Northern Cape Province, South Africa). *African Archaeological Review*, 32: 595–612

Klein, R. G., Cruz-Uribe, K. & Beaumont, P. B. 1991. Environmental, ecological, and paleoanthropological implications of the late Pleistocene mammalian fauna from Equus Cave, Northern Cape Province, South Africa. *Quaternary Research*, 36: 94–119.

Latham, A. G., McKee, J. K. & Tobias, P. V. 2007. Bone breccias, bone dumps, and sedimentary sequences of the western Limeworks, Makapansgat, South Africa. *Journal of human evolution*, 52: 388–400.

Laycock, P. 1973. Distribution and abundance of bats in the Natal Midlands (Mammalia: Chiroptera). *Annals of the Transvaal Museum*, 28: 207–227.

Matthews, T. 1999. Taphonomy and the micromammals from Elands Bay Cave. *The South African Archaeological Bulletin*, 133–140.

McDonald, J. T., Rautenbach, I. L. & Nel, J. A. J. 1990. Foraging ecology of bats observed at De Hoop Provincial Nature Reserve, southern Cape Province. *South African Journal of Wildlife Research*, 20: 133–145.

Miller-Butterworth, C. M., Jacobs, D. S. & Harley, E. H. 2003. Strong population substructure is correlated with morphology and ecology in a migratory bat. *Nature*, 424: 187–191.

Miller, C. E., Goldberg, P. & Berna, F. 2013. Geoarchaeological investigations at Diepkloof Rock Shelter, Western Cape, South Africa. *Journal of Archaeological Science*, 40: 3432–3452.

Moir, M. & Durand, J. 2012. Omgewingsfaktore wat die hiberneringsgedrag van die Natalse langvinger-vlermuis (*Miniopterus natalensis*) in die Bakwena-grot, Gauteng, beïnvloed. *Suid-Afrikaanse Tydskrif vir Natuurwetenskap en Tegnologie*, 31: 1.

Mutumi, G. L., Jacobs, D. S. & Winker, H. 2016. Sensory drive mediated by climatic gradients partially explains divergence in acoustic signals in two horseshoe bat species, *Rhinolophus swinnyi* and *Rhinolophus simulator*. *PloS one*, 11: e0148053.

Nel, T. H., Wurz, S. & Henshilwood, C. S. 2018. Small mammals from marine isotope stage 5 at Klasies River, South Africa-reconstructing the local palaeoenvironment. *Quaternary International*, 471: 6–20.

Overal, W. & Wingate, L. 1976. The biology of the batbug *Stricticimex antennatus* (Hemiptera: Cimicidae) in South Africa. *Annals of the Natal Museum*, 22: 821–828.

Paweska, J. T., van Vuren, P. J., Kemp, A., Storm, N., Grobbelaar, A. A., Wiley, M. R., Palacios, G. & Markotter, W. 2018. Marburg Virus Infection in Egyptian Rousette Bats, South Africa, 2013-2014. *Emerging Infectious Diseases*, 24: 1134–1137.

Plug, S. & Badenhorst, S. 2001. The distribution of macromammals in Southern Africa over the past 30 000 Years: as reflected in animal remains from archaeological sites. Present and historical distributions of macromammals in southern Africa. Order- Artiodactyla. *Transvaal Museum Monographs*, 12: 1–234.

Salata, H. A. B. 2012. Environmental factors influencing the distribution of bats (Chiroptera) in South Africa. PhD Thesis. University of Cape Town, South Africa.

Schubert, B. W., Ungar, P. S., Sponheimer, M. & Reed, K. E. 2006. Microwear evidence for Plio-Pleistocene bovid diets from Makapansgat Limeworks Cave, South Africa. *Palaeogeography, Palaeoclimatology, Palaeoecology*, 241: 301–319.

Staegemann, M. W. 2016. Drivers of bat fly diversity and prevalence of six Rhinolophus bat species in southern Africa. PhD Thesis. University of Kwazulu-Natal, South Africa.

Taylor, P., Cheney, C. & Sapsford, C. 1999. Roost habitat evaluation and distribution of bats (Chiroptera) in the Durban Metropolitan Region. *Durban Museum Novitates*, 24: 62–71.

Van der Merwe, M. 1973. Aspects of temperature and humidity in preferred hibernation sites of the Natal clinging bat *Miniopterus schreibersi natalensis* (A. Smith, 1834). *African Zoology*, 8: 121–134.

Van der Merwe, M. 1975. Preliminary study on the annual movements of the Natal clinging bat. *South African Journal of Science*, 71: 237–241.

Van der Merwe, M. 1978. Post-natal development and mother-infant relationships in the Natal clinging bat *Miniopterus schreibersi natalensis* (A. Smith, 1834). In: *Proceedings of the Fourth International Bat Research Conference*, pp. 309–322.

Van der Merwe, M. 1980. Importance of *Miniopterus schreibersi natalensis* in the diet of barn owls. *South African Journal of Wildlife Research*, 10: 15–17.

Verkerk, V. 2017. The potential of Wonderboom Nature Reserve as an archaeotourism destination. PhD Thesis. University of Pretoria, South Africa.

Wood, S. 2012. Geographic distribution and composition of the parasite assemblage of the insectivorous bat, *Miniopterus natalensis* (Chiroptera: Miniopteridae), in South Africa. PhD Thesis. University of Cape Town, South Africa.
